# The mechanical uncoupler blebbistatin is associated with significant electrophysiological effects in the isolated rabbit heart

**DOI:** 10.1113/expphysiol.2012.069369

**Published:** 2013-01-04

**Authors:** Kieran E Brack, Ravi Narang, James Winter, G André Ng

**Affiliations:** 1 Cardiology group, Department of Cardiovascular Sciences, University of LeicesterLeicester LE3 9QP, UK; 2 Leicester NIHR Biomedical Research Unit in Cardiovascular Disease, Glenfield HospitalLeicester LE3 9QP, UK

## Abstract

Blebbistatin (BS) is a recently discovered inhibitor of the myosin II isoform and has been adopted as the mechanical uncoupler of choice for optical mapping, because previous studies suggest that BS has no significant cardiac electrophysiological effects in a number of species. The aim of this study was to determine whether BS affects cardiac electrophysiology in isolated New Zealand White rabbit hearts. Langendorff-perfused hearts (*n*= 39) in constant-flow mode had left ventricular monophasic action potential duration (MAPD) measured at apical and basal regions during constant pacing (300 ms cycle length). Standard action potential duration restitution was obtained using the single extrastimulus method with measurement of the maximal restitution slope. Ventricular fibrillation threshold was measured as the minimal current inducing sustained ventricular fibrillation with burst pacing (30 stimuli, at 30 ms intervals). Optical action potentials were recorded using the voltage-sensitive dye di-4-ANEPPS. Measurements were taken at baseline and after 60 min perfusion with BS (5 μm). Blebbistatin significantly prolonged left ventricular apical (mean ± SEM; from 129.9 ± 2.9 to 170.7 ± 4.1 ms, *P* < 0.001, *n*= 8) and basal MAPD (from 135.0 ± 2.3 to 163.3 ± 5.6 ms, *P* < 0.001) and effective refractory period (from 141.3 ± 4.8 to 175.6 ± 3.7 ms, *P* < 0.001) whilst increasing the maximal slope of restitution (apex, from 0.79 ± 0.09 to 1.57 ± 0.16, *P* < 0.001; and base, from 0.71 ± 0.06 to 1.44 ± 0.24, *P* < 0.001) and ventricular fibrillation threshold (from 5.3 ± 1.1 to 17.0 ± 2.9 mA, *P* < 0.001). In other hearts, blebbistatin significantly prolonged optically recorded action potentials (from 136.5 ± 6.3 to 173.0 ± 7.9 ms, *P* < 0.05, *n*= 4). In control experiments, the increase of MAPD with blebbistatin was present whether the hearts were perfused in constant-pressure mode (*n*= 5) or in unloaded conditions (*n*= 5). These data show that blebbistatin significantly affects cardiac electrophysiology. Its use in optical mapping studies should be treated with caution.

**New Findings**

- What is the central question of this study?
  Does the mechanical uncoupler blebbistatin affect cardiac electrophysiology?
- What is the main finding and what is its importance?
  Blebbistatin significantly affects cardiac ventricular electrophysiology and induction of ventricular fibrillation. This is a new finding that has serious implications for optical mapping studies where blebbistatin is used to inhibit cardiac contraction.

Optical mapping techniques for monitoring cardiac membrane potential are growing in popularity in cardiac electrophysiology research and are the technique of choice for elucidating the mechanisms that underlie cardiac arrhythmias. Optical mapping employs the use of a voltage-sensitive dye to allow the non-invasive, simultaneous recording of membrane voltage from multiple sites, and in the cardiac ventricle this can encompass the entire left ventricular (LV) epicardial surface, providing detailed spatial and temporal measurements of electrical activity. A major challenge in optical mapping is motion artefact generated from myocardial contraction, which results in a low signal-to-noise ratio (SNR) and poor quality action potential (AP) recordings. Several methods have been used to overcome this obstacle, ranging from mechanical techniques where the heart is contained within a glass chamber to reduce contractile movement (Salama *et al.* 1987; Efimov *et al.* 1996) to pharmacological methods, in which an excitation–contraction uncoupler (ECU) is used to eliminate myocardial contraction. 2,3-Butanedione monoxime and cytochalasin-D are two ECUs with effective uncoupling properties in cardiac models (Biermann *et al.* 1998; Sakai, 2006; Jou *et al.* 2010) and were historically used in optical mapping. However, significant effects on cardiac electrophysiology have been reported (Blanchard *et al.* 1990; Liu *et al.* 1993; Maesako *et al.* 2000; Watanabe *et al.* 2001; Baker *et al.* 2004; Kettlewell *et al.* 2004), which limit their use in optical mapping.

Blebbistatin (BS) is a recently discovered molecule that has been reported as an effective ECU in cardiac preparations (Dou *et al.* 2007; Fedorov *et al.* 2007; Jou *et al.* 2010) and is now the mechanical uncoupler of choice in optical mapping of cardiac electrophysiology. Blebbistatin inhibits the myosin II–ADP-phosphate complex of skeletal muscle and non-muscle myosin II isoforms, whilst in its free state, limiting further interaction with actin filaments (Limouze *et al.* 2004). Its behaviour in this non-bound state is advantageous because it prevents any stiffness, which may result from a constant actin–myosin cross-link (Farman *et al.* 2008). Its effectiveness as an ECU with minimal effects on cardiac electrophysiology has been reported in various experimental preparations in different species (Dou *et al.* 2007; Fedorov *et al.* 2007; Farman *et al.* 2008; Jou *et al.* 2010), including cardiac tissue from the zebra fish, mouse, rat and rabbit, suggesting that blebbistatin should be the ideal ECU for use in optical mapping studies.

The effect of blebbistatin on several electrophysiological parameters has been measured; however, to our knowledge, the effects of BS on perfusion pressure, the electrical restitution property of the heart and inducibility of ventricular fibrillation (VF) have not been adequately examined. The action potential duration (APD) restitution property of the myocardium is considered to be a key factor in arrhythmogenesis with increased wavebreak, oscillations and susceptibility to VF, and the importance of this property has been previously reviewed (Karma, 1994; Weiss *et al.* 2000). Measuring the effect of blebbistatin on electrical restitution and VF is therefore important in order to justify its continued use in cardiac arrhythmia research.

Evidence suggests that an APD restitution curve with a maximal gradient >1 increases the likelihood of oscillations and the break-up of spiral waves or ‘mother rotors’ into multiple wavelets, which is a key process in the initiation of VF (Cao *et al.* 1999). Further evidence suggests that drugs or manoeuvres that reduce the slope of the APD restitution curve <1 lead to a reduction in VF vulnerability (Garfinkel *et al.* 2000). The spatial heterogeneity of electrical restitution has rarely been reported and modern optical mapping techniques provide a novel opportunity to assess its influence on arrhythmia susceptibility. In order to ensure that the electrical restitution data collected from optical mapping studies are comparable with the data already collected from non-optical studies, it is vital that any ECU in use does not have a direct effect on the restitution property of the myocardium or, indeed, on arrhythmia inducibility. The aim of the present study was to use non-optical mapping techniques to measure the effects of blebbistatin on ventricular electrophysiological properties in isolated, Langendorff-perfused rabbit hearts.

## Methods

### Ethical approval

All procedures were undertaken after local ethics approval at the University of Leicester and were carried out in accordance with the UK Animals (Scientific Procedures) Act 1986 and the *Guide for the Care and the Use of Laboratory Animals* published by the US National Institutes of Health (NIH publication no. 85-23, revised 2010).

### Isolation of rabbit hearts

Adult male New Zealand White rabbits (2.8 ± 0.1 kg, *n*= 39; Harlan, Shardlow, UK) were sedated with an intramuscular injection containing a mixture of medetomidine hydrochloride (0.2 mg kg^−1^; Sedator; Dechra, Shrewsbury, UK), ketamine (Narketan, Fort Dodge, Southampton, UK; 10 mg kg^−1^) and butorphanol (Torbugesic, Fort Dodge, Southampton, UK; 0.05 mg kg^−1^). Following 15–20 min of sedation, the animals were heparinized (1000 IU; Multiparin, Wrexham, UK) and then killed with an overdose of pentobarbitone sodium (160 mg kg^−1^; Sagatal; Rhone Merieux, Harlow, UK) administered via the marginal ear vein. Hearts were rapidly excised and briefly immersed in ice-cold Tyrode solution before cannulation via the ascending aorta and perfusion in a retrograde Langendorff manner with Tyrode solution of the following composition (mm): Na^+^, 138.0; K^+^, 4.0; Ca^2+^, 1.8; Mg^2+^, 1.0; HCO_3_^−^, 24.0; H_2_PO_4_^−^, 0.4; Cl^−^, 121; glucose, 11; and acetate, 20.0. The solution was warmed to 37°C and was kept at a pH of 7.4 by continuous bubbling with a 95% O_2_–5% CO_2_ gas mixture. The time from death to cannulation was within 90 s. Hearts were perfused in a single-pass non-recirculating manner to avoid the confounding effects of byproducts that accumulate from perfusion effluent. A 3 French catheter (Portex, Kent, UK) was inserted through the left ventricular apex to drain Thebesian venous effluent. Left ventricular pressure (LVP) was monitored with a fluid-filled latex balloon inserted into the left ventricle via the left atrium. The balloon was connected to a pressure transducer (MTL0380; ADInstruments Ltd, Chalgrove, UK) using a 3 French cannula and the volume adjusted to keep end-diastolic pressure at 0–5 mmHg. Perfusion pressure (PP) was monitored with a second pressure transducer connected in series with the aortic cannula.

### Cardiac electrical recording and pacing

The techniques for measuring monophasic action potentials, standard APD restitution and ventricular fibrillation threshold (VFT) have been reported before (Brack *et al.* 2007, 2011; Ng *et al.* 2007). These are described in brief as follows.

Quadripolar contact electrodes (EP Technologies, Sunnyvale, CA, USA) were used to record monophasic action potentials (MAP) at the apical and basal epidcardial surfaces of the left ventricle using a custom-made DC-coupled high-input-impedance differential amplifier (Biomedical Joint Workshop, University of Leicester, UK; Brack *et al.* 2007, 2011; Ng *et al.* 2007). Two pairs of platinum hook electrodes (Grass Instruments, Astro-Medical Inc., Slough, UK) were attached to the right atrial appendage; one pair was used for recording right atrial electrograms and the other pair for atrial pacing. Ventricular pacing was established using a bipolar catheter (EP Technologies) inserted into the right ventricular apex, at twice the diastolic threshold, using a 2 ms pulse width with a constant-current stimulator (DS7A; Digitimer, Welwyn Garden City, UK).

### Measurements

#### Atrioventricular conduction

Atrioventricular conduction time was measured during atrial pacing (cycle length 300 ms, over 5 min steady state) from the atrial stimulus spike to the time of activation of the ventricular MAP recorded at the apical region and the basal region.

#### Ventricular monophasic action potential duration (MAPD)

Hearts were paced at the right ventricular apex at the following three different cycle lengths: 300, 250 and 200 ms, for 5 min steady state at each cycle length. The MAPD was measured from the time of MAP activation to repolarization at 90% decay. The interval from the time of the ventricular stimulus spike to the activation of the MAP was also measured. Monophasic action potential duration and all measurements were made from MAPs at both apical and basal regions using NewMap software (Dr Francis Burton, University of Glasgow, Glasgow, UK).

#### Action potential duration restitution protocol

Standard APD restitution data were obtained with right ventricular pacing using a 20 beat drive train (S1) at 300 ms cycle length (CL) followed by an extrastimulus (S2), and repeated with progressively shorter S1–S2 intervals [by 10 ms from 300 to 200 ms and by 5 ms from 200 ms to the effective refractory period (ERP), defined as the longest S1–S2 interval which failed to capture the ventricle; Brack *et al.* 2007, 2011; Ng *et al.* 2007]. Timings of the beginning of the S1 and S2 MAP signals were noted and the delays from the pacing stimuli measured. A custom-written program (NewMap; Dr Francis Burton) was used to measure MAPD from the beginning of the MAP signal to 90% repolarization (MAPD_90_). Restitution was examined by analysis of the relationship between S2-MAPD_90_ and preceding diastolic intervals (DI, defined as the interval between the S1 and S2 MAP signals minus S1-MAPD_90_). An exponential curve [MAPD_90_= MAPD_90max_ (1 – e^−DI/τ^)] was fitted using Microcal Origin Pro (version 8.07; Origin, San Diego, CA, USA), where MAPD_90max_ is the maximal MAPD_90_ and τ the time constant. The maximal slope of restitution was obtained by analysing the first derivative of the fitted curve.

#### Ventricular fibrillation threshold protocol

Ventricular fibrillation threshold (VFT) data were obtained with right ventricular pacing using a 20 beat drive train at 300 ms CL followed by a rapid 30 beat train at 30 ms CL with a pacing current of 0.5 mA. Following a 5 s rest period, this was repeated with 0.5 mA increments in pacing current until the induction of sustained VF. The VFT was defined as the minimal current required to induce sustained VF, i.e. VF that required cardioversion (Brack *et al.* 2007, 2011; Ng *et al.* 2007). After VF was induced, a 2–4 ml bolus of KCl (50 mm) was injected into the perfusion line for cardioversion. A period of 15 min was followed to allow the heart to return to baseline.

### Experimental protocols

#### Time-based stability

In order to establish stability of recorded parameters, left ventricular pressure (LVP), perfusion pressure (PP), effective refractory period (ERP), ventricular fibrillation threshold (VFT), monophasic action potential duration (MAPD) and the maximal slope of the electrical restitution curve recorded at apical and basal sites were recorded at hourly intervals for a 5 h period.

#### Effect of blebbistatin

After a stabilization period of 30 min, the atrial pacing protocol, ventricular pacing protocols at each cycle length, standard APD restitution and VFT protocols were carried out in the following conditions: (i) baseline; (ii) after 60 min perfusion with blebbistatin (5 μm;± enantiomer; Tocris Bioscience, Abingdon, UK); and (iii) 2 h after returning to Tyrode solution without BS (washout). Blebbistatin was prepared fresh on the day of the experiment, by dissolving in DMSO (2 mg ml^−1^), and subsequently added directly into the Tyrode solution at 37°C. This resulted in an overall DMSO concentration of 0.0014% (v/v). All experiments were conducted in the dark. In seven hearts, a single bipolar ECG was recorded (ADInstruments bioamplifier ML 136) using two 3.5-mm-tipped catheters (Biosense Webster, Diamond Bar, CA, USA) secured on the inner surface of a temperature-controlled glass organ bath, in which the hearts were immersed in Tyrode solution.

### Perfusion and loading conditions

The main experimental data set was obtained with hearts perfused in our standard perfusion mode, i.e. with a constant flow of 45 ml min^−1^ and in the presence of an intraventricular fluid-filled balloon adjusted to give a diastolic pressure of 0–5 mmHg (*n*= 8).

#### Control experiments

In all control experiments, measurements were performed using our modified Tyrode solution (details above; see ‘*Isolation of rabbit hearts*’) and the Tyrode solution of Fedorov *et al.* (2007) containing (mm): Na^+^, 150.6; K^+^, 4.7, Ca^2+^, 1.3; Mg^2+^, 1.05; HCO_3_^−^, 20.0; H_2_PO_4_^−^, 1.19; Cl^−^, 135.5; and glucose, 11. All solutions were perfused through an inline Micropore prefilter and 5 μm filter (Millpore, Watford, UK) using a Gilson Minipuls 3 peristaltic pump (Anachem, Luton, UK) with the perfusion mode controlled using an STH pump controller (model ML175; ADInstruments Ltd). The control experiments were carried out to address the following questions.

- Is MAPD affected by constant-flow *versus* constant-pressure Langendorff mode? Hearts were initially perfused at steady state in constant-flow mode, then switched to constant-pressure mode with pressure matched to that measured during constant flow (*n*= 5). The effect on MAPD of changing from constant flow to constant pressure was measured during ventricular pacing at 300 ms (*n*= 5).
- Does blebbistatin affect MAPD with an unloaded left ventricle? The effect of blebbistatin on ventricular MAPD was observed after deflating the LV intraventricular balloon (*n*= 5). The left ventricle was therefore unloaded. Hearts remained in constant-flow Langendorff perfusion, and MAPD was measured during ventricular pacing at 300 ms.
- Does blebbistatin affect MAPD in constant-pressure Langendorff mode with an unloaded left ventricle? Hearts were perfused in constant-perfusion Langendorff mode with an unloaded left ventricle (*n*= 5), and the effect of blebbistatin on MAPD was measured during ventricular pacing at 300 ms.
- What is the dose–response relationship?In six hearts, the dose–response relationship of the effect of blebbistatin was tested. After hearts had stabilized for a minimal period of 30 min, incremental doses of blebbistatin were perfused at 0.5, 1, 5 and 10 μm. Left ventricular pressure, perfusion pressure and MAPD were measured, and data are reported at steady state.

### Optical mapping

Action potentials were measured after a bolus injection of di-4-ANEPPS (Mantravadi *et al.* 2007; 40 μl, 1 mg ml^−1^ in DMSO; Invitrogen, UK). Previously published data (Ogura *et al.* 1995; Biermann *et al.* 1998) suggest that DMSO is detrimental for electrophysiology at concentrations of 1.5% (w/v) and above. The calculated concentration used in our study is below 0.1%, which makes it unlikely that its use is detrimental. Light from an LED light source (535 nm) was projected onto the heart using a 570 nm dichroic mirror, with emitted light collected through a 630 nm long-pass filter onto a Hamamatsu 16 × 16 element photodiode array (Cairn Research, Faversham, UK). Selected pixels were output from the PDA camera and recorded as described below, for simultaneous recording along with the physiological parameters for monitoring change during the experiment. For recording specific sections of optical data at 256 sites, QRecord software (Dr Francis Burton, University of Glasgow) was used and digitized data at 2000 frames s^−1^. Once recorded, QRecord also allows files to be converted into a format that was analysed in ADInstruments Chart software.

### Signal measurements and statistical analysis

All signals were recorded with a PowerLab 16/30 system (ADInstruments Ltd) and digitized at 1 kHz using Chart and Scope software (ADInstruments Ltd), with the data stored and displayed on a personal desktop computer. All data are expressed as means ± SEM. Optical action potentials were analysed with Peak Parameters and Scope software within Chart software. Statistical analysis was carried out using a one-way ANOVA with Tukey *post hoc* test or Student's paired *t* test for comparisons where appropriate. A *P* value <0.05 was considered significant.

## Results

### Time-based control data

**Figure 1:**
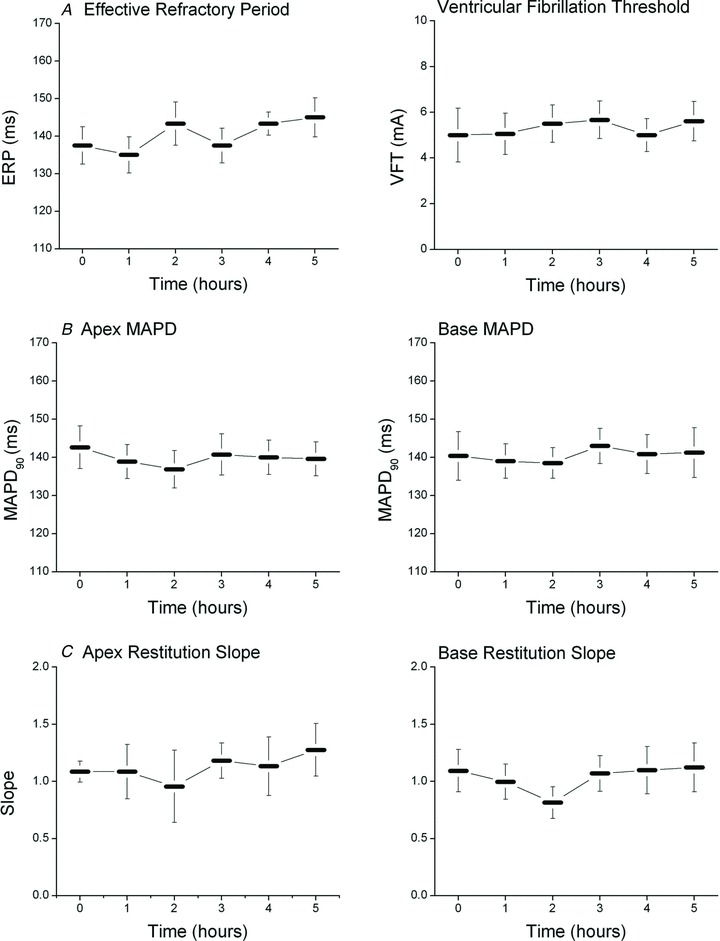
Time-based control data Mean data illustrating repeated measurements of effective refractory period (ERP) and ventricular fibrillation threshold (*A*), monophasic action potential duration (MAPD) from the apex and base (*B*) and maximal slope of the electrical restitution curve at apical and basal sites (*C*) over a 5 h time period in the isolated, Langendorff-perfused rabbit heart (*n*= 8).

In order to establish stability of our preparation over time, we recorded left ventricular pressure, perfusion pressure, effective refractory period, ventricular fibrillation threshold, monophasic action potential duration and the maximal slope of the electrical restitution curve at apical and basal sites at hourly intervals for a 5 h period (*n*= 8). Left ventricular pressure slowly decreased over a 5 h period by ∼15% (from 86.2 ± 2.4 to 71.1 ± 7.5 mmHg, *P* < 0.001), whilst PP (from 62.4 ± 3.9 to 63.3 ± 5.4 mmHg, *P* > 0.05) and heart rate (HR; from 152.9 ± 9.5 to 137.7 ± 7.3 beats min^−1^, *P* > 0.05) did not change significantly. With respect to the electrophysiological parameters, there was no significant (*P* > 0.05) change in ERP, VFT, MAPD or electrical restitution slope (Fig. 1) over the course of the experiment.

### The effect of blebbistatin during constant-flow Langendorff mode with a loaded left ventricle

**Figure 2:**
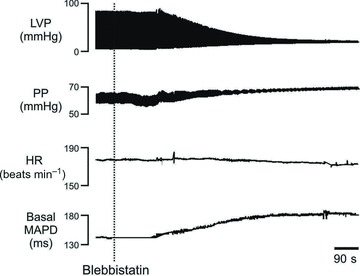
The effect of blebbistatin in the isolated rabbit heart Raw data on the dynamic effect of blebbistatin (5 μm) on left ventricular pressure (LVP), perfusion pressure (PP), heart rate (HR) and monophasic action potential duration (MAPD) from the basal region in the isolated, Langendorff-perfused rabbit heart.

**Figure 3:**
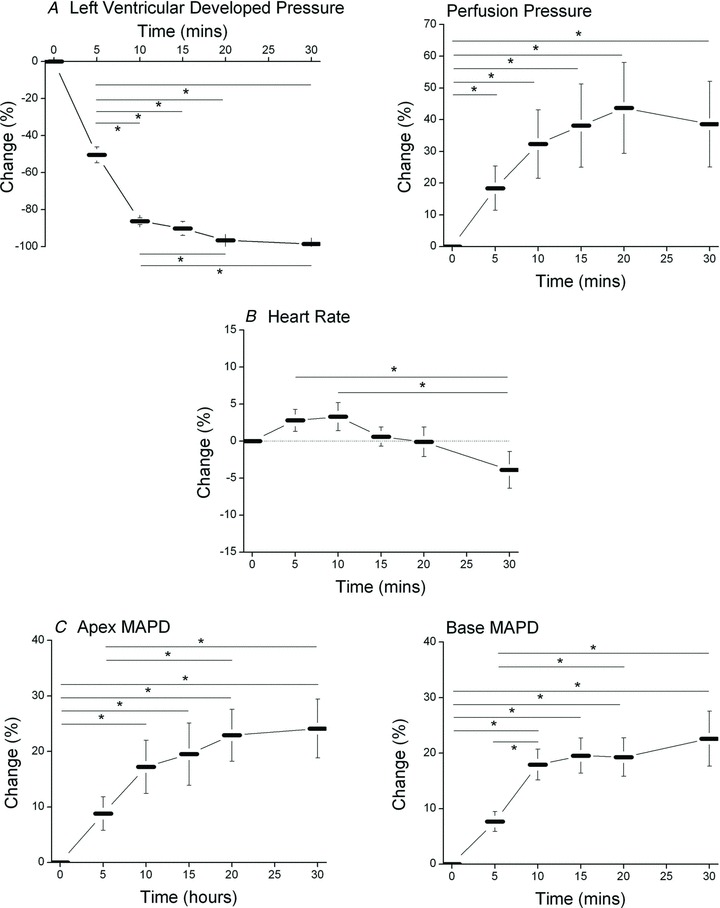
The dynamic effect of blebbistatin on cardiac physiology Mean data showing the percentage change in left ventricular developed pressure and perfusion pressure (*A*), heart rate (*B*) and MAPD recorded from apical and basal sites (*C*) over the first 30 min perfusion with 5 μm blebbitstatin. *P < 0.05 as indicated by the horizontal bars, *n*= 8.

Raw data illustrating the effect of blebbistatin on LVP, PP, HR and MAPD are shown in Fig. 2. As expected, blebbistatin led to a gradual decrease in LVP, and left ventricular developed pressure was abolished within 20 min. After 60 min perfusion with blebbistatin, systolic LVP decreased significantly from a baseline of 88.0 ± 6.3 to 18.8 ± 2.9 mmHg (*P* < 0.001, *n*= 8), whilst end-diastolic pressure increased from 4.8 ± 0.6 to 18.4 ± 2.9 mmHg (*P* < 0.001, *n*= 8). The resultant developed pressure was therefore abolished from 83.2 ± 6.4 to 0.4 ± 0.2 mmHg (*P* < 0.001, *n*= 8). Concerning perfusion pressure, there was a biphasic response, with an initial and significant vasodilatation at 1 min perfusion (from 63.5 ± 6.5 to 59.2 ± 2.2 mmHg, *P* < 0.05), followed by a secondary and steady increase in pressure to 97.9 ± 9.9 mmHg (*P* < 0.01, *n*= 8) following 60 min of blebbistatin perfusion. Heart rate was unchanged from a baseline of 160.7 ± 4.9 to 154.0 ± 4.9 beats min^−1^ following blebbistatin (*P* > 0.05, *n*= 8). After a 2 h washout period, LV systolic and LV developed pressure returned to a level that was significantly lower than baseline (50.0 ± 4.1 and 45.1 ± 3.9 mmHg, respectively, *P* < 0.001, *n*= 8), whilst EDP returned back to baseline 4.8 ± 1.3 mmHg). Perfusion pressure remained significantly higher than baseline (87.4 ± 6.5 mmHg, *P* < 0.05, *n*= 8). Figure 3 illustrates the percentage change in each parameter during the first 30 min of perfusion with blebbistatin. Compared with control conditions, there is a significant (*P* < 0.05) change in LVP and PP within 5 min perfusion, and within 10 min for MAPD at both regions. Values begin to stabilize after 10 min perfusion with blebbistatin, indicating that changes in cardiac parameters occur from the beginning of perfusion.

#### Effect of blebbistatin on atrioventricular conduction

There was no significant effect of blebbistatin on atrioventricular conduction (apical MAP atrioventricular conduction, from 111.2 ± 5.9 to 110.4 ± 4.7 ms; and basal MAP atrioventricular conduction, from 107.0 ± 5.3 to 109.2 ± 6.0 ms, *P* > 0.05, *n*= 5). The threshold for atrial capture did not change during perfusion with blebbistatin (from 0.23 ± 0.04 to 0.21 ± 0.07 mA).

#### Effect of blebbistatin on ventricular MAPD

**Figure 4:**
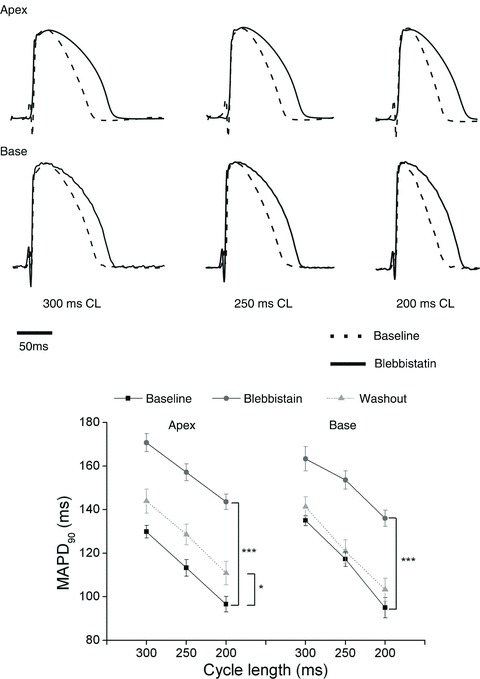
Effect of blebbistatin on MAPD Top panel shows amplitude-normalized monophasic action potentials (MAPs) recorded from the apical (top traces) and basal region (bottom panel) of the left ventricle at baseline (dashed trances) and after 60 min of blebbistatin (continuous traces) during right ventricular pacing at cycle lengths (CL) of 300, 250 and 200 ms. Bottom panel, mean data showing MAPD_90_ at apical and basal sites at baseline, following 60 min perfusion with blebbistatin and following a 2 h washout period. *P < 0.05, ***P < 0.001, *n*= 8.

Raw and mean data illustrating the effect of blebbistatin on LV MAPs recorded from the apical and basal regions at each ventricular pacing CL studied are shown in Fig. 4, illustrating that there was a significant rate-dependent shortening of MAPD at baseline, during blebbistatin perfusion and during washout (*P* < 0.001). Blebbistatin caused a significant prolongation of apical MAPD at all cycle lengths studied. The MAPD was increased by 31.8 ± 3.9% (40.9 ± 4.5 ms), 39.4 ± 5.0% (43.9 ± 4.3 ms) and 50.0 ± 7.1% (47.0 ± 5.0 ms) at the apex and by 21.0 ± 4.0% (28.3 ± 5.3 ms), 31.5 ± 4.8% (36.3 ± 4.7 ms) and 45.2 ± 7.9% (41.0 ± 5.2 ms) at the base at cycle lengths of 300, 250 and 200 ms, respectively. The threshold for ventricular capture did not change with blebbistatin (from 0.18 ± 0.03 to 0.21 ± 0.04 mA).

#### Effects of blebbistatin on ERP, VFT and standard APD restitution

The effects of blebbistatin on ERP, VFT and standard APD restitution were measured. Blebbistatin significantly increased both ERP (from 141.3 ± 4.8 to 175.6 ± 3.7 ms, *P* < 0.001) and VFT (from 5.3 ± 1.1 to 17.0 ± 2.9 ms, *P* < 0.001). Both ERP (160.6 ± 7.8 ms) and VFT (18.1 ± 3.6 mA) values failed to return to baseline after washout.

**Figure 5:**
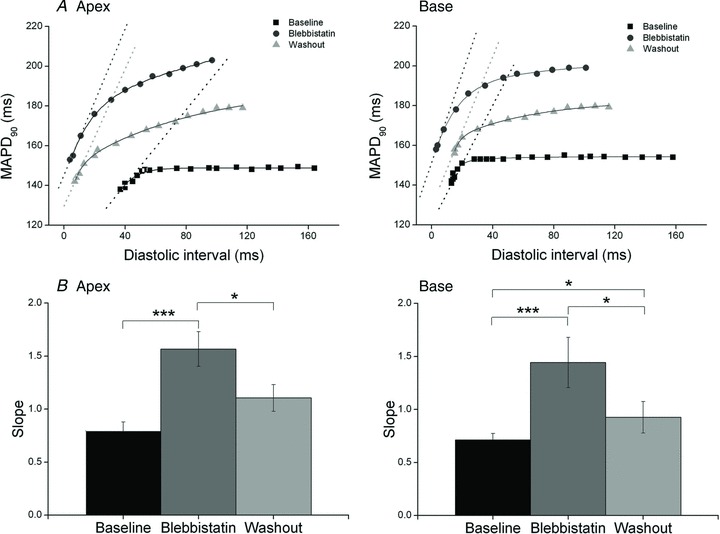
Effect of blebbistatin on action potential duration (APD) restitution *A*, plot of APD restitution curves from a typical experiment at baseline, following 60 min of blebbistatin and following a 2 h washout period at apical (left) and basal regions (right), with the dotted lines representing the maximal slopes. Each symbol represents the APD at each S1–S2 interval, whilst the continuous line represents the fitted single exponential curve. *B*, mean data illustrating maximal restitution slope (Slope) at baseline, during perfusion with blebbistatin and after a 2 h period of washout at apal (left) and basal sites (right). *P < 0.05, ***P < 0.001, *n*= 8.

The effects of blebbistatin on APD restitution and the maximal slope of restitution are illustrated in Fig. 5. Each symbol in Fig. 5 represents the APD at each S1–S2 interval, whilst the continuous line is the fitted single exponential curve. Maximal MAPD_90_ was significantly (*P* < 0.001) increased from a baseline of 139.2 ± 3.2 ms (apex) and 135.5 ± 2.7 ms (base) to 174.3 ± 4.4 ms (apex) and 167.4 ± 4.5 ms (base). Following washout, values were still significantly higher than baseline [170.4 ± 4.9 ms (apex) and 165.5 ± 4.7 ms (base); *P* < 0.001, baseline *versus* washout, *n*= 8]. Blebbistatin significantly increased the maximal restitution slope, as indicated by the dotted lines in Fig. 5*A*. Mean data in Fig. 5*B* confirm this finding. Following washout, the maximal slope values were significantly higher than those obtained at baseline.

**Figure 6:**
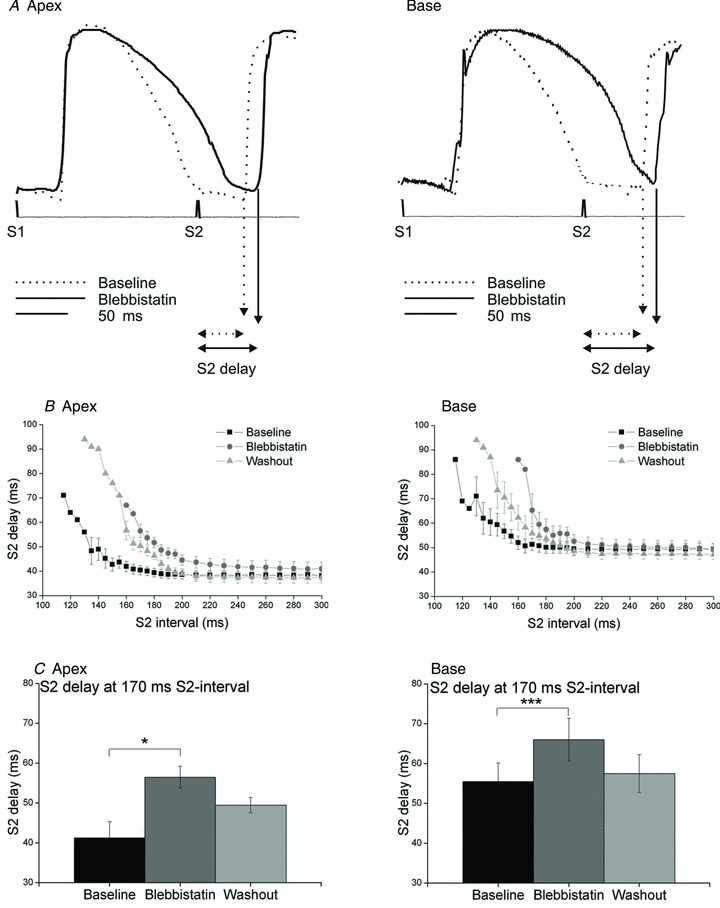
Effect of blebbistatin on S2 delay *A*, raw data illustrating monophasic action potentials recorded at apical (left) and basal regions (right) at the S1–S2 coupling interval of 170 ms at baseline and during perfusion with blebbistatin. *B*, mean data (*n*= 8) illustrating S2 delay at baseline, after 60 min perfusion with blebbistatin and after a 2 h period of washout at apical (left) and basal regions (right). *C*, S2 delay at a selected S2 interval of 170 ms (*n*= 4).*P < 0.05, ***P < 0.001.

During the extrastimulus protocol, the delay from S2 to the beginning of the S2 MAP signal (S2 delay) was measured and increased with progressively shorter S1–S2 intervals. An illustration of S2 delay is shown in Fig. 6*A*. This prolongation in S2 delay suggests a slowing of conduction at short coupling intervals that would be consistent with restitution of conduction velocity (Cao *et al.* 1999). Figure 6 shows mean data on the effects of blebbistatin on S2 delay over the range of S1–S2 intervals. For comparison, the mean S2 delay at an S1–S2 interval of 170 ms increased significantly at both apex and basal regions (Fig. 6*B* and *C*, *P* < 0.05). Although the exact conduction path of the paced beats was not mapped, hence conduction velocity not measured directly, these data suggest that conduction velocity at short diastolic intervals is decreased with blebbistatin.

#### Effect of blebbitstatin on QT interval on ECG

**Figure 7:**
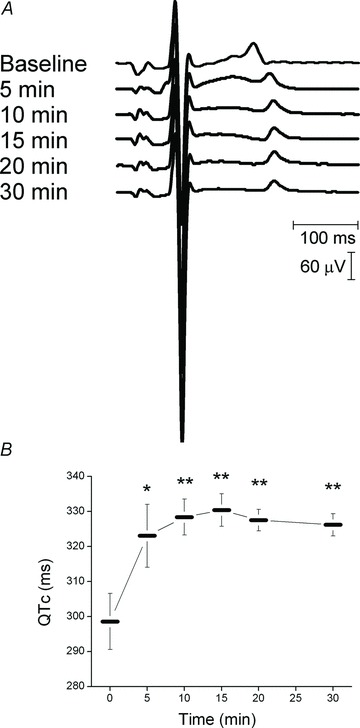
The effect of blebbistatin on the ECG *A*, aligned and averaged ECGs. *B*, mean data recorded at baseline and at specific time points during perfusion with 5 μm blebbistatin. *n*= 7, *P < 0.05, **P < 0.01 *versus* time 0.

Figure 7*A* shows the raw data (*n*= 7) on changes in ECG recorded during perfusion with blebbistatin. There was no significant ST segment shift at baseline or during blebbistatin perfusion. Blebbistatin caused a significant increase in QT interval by 5 min perfusion, reaching a plateau between 10 and 15 min, mirroring the changes in MAPD (Fig. 3). Mean data on QT interval corrected for the non-significant changes in HR (QTc; Bazett's formula) are shown in Fig. 7*B*.

### Control experiments

#### Constant flow *versus* constant pressure

**Figure 8:**
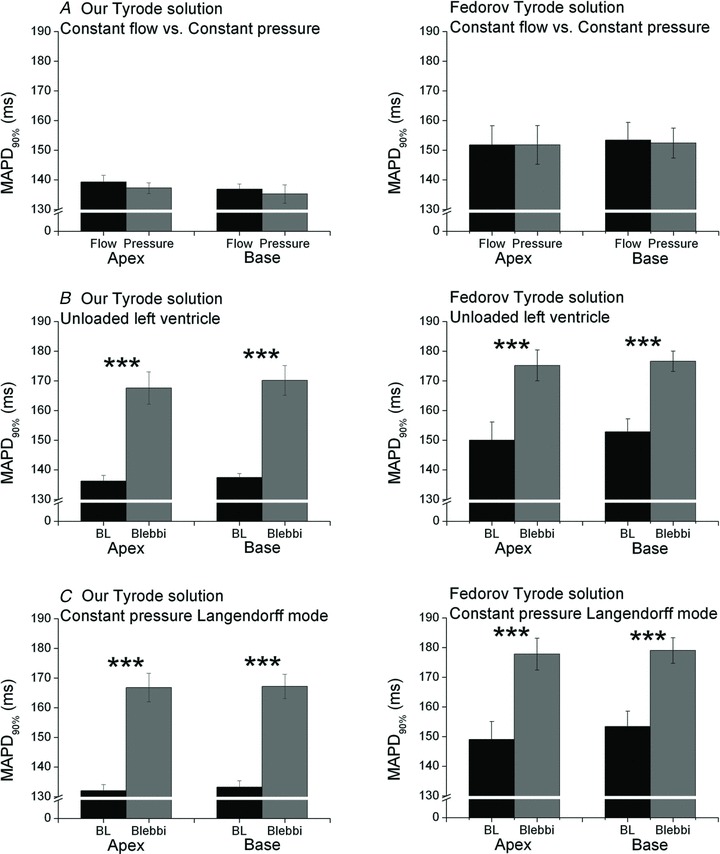
Control data Mean data illustrating the effect of changing between constant-flow to constant-pressure Langendorff perfusion on MAPD (*A*), the effect of blebbistatin (Blebbi) perfusion on MAPD in hearts without any intraventricular load (unloaded; *B*) and the effect of blebbistatin in unloaded hearts during constant-pressure Langendorff perfusion (*C*). Experiments were conducted in our modified Tyrode solution (left panels) and in the Tyrode solution used by Fedorov *et al.* (2007; right panels; *n*= 5 in all groups). ***P < 0.001 *versus* baseline (BL)

The effects of changing from constant-flow to constant-pressure Langendorff mode using our modified Tyrode solution and that of Fedorov *et al.* (2007) are illustrated in Fig. 8*A* (*n*= 5 for each group). There was a trend for MAPD to be longer using the Tyrode solution of Fedorov *et al.* (2007), but this failed to reach significance (between *P*= 0.05 and *P*= 0.06). However, irrespective of the Tyrode solution used, changing from constant-flow to constant-pressure mode did not significantly (*P* > 0.05) affect MAPD.

#### The effect of blebbistain in ‘unloaded’ hearts

The effects of blebbistatin in hearts that did not have any intraventricular load (deflated balloon; ‘unloaded’) using our modified Tyrode solution and that of Fedorov *et al.* (2007) are illustrated in Fig. 8*B* (*n*= 5 for each group). The MAPD was significantly (*P* < 0.001) increased during blebbistatin perfusion irrespective of the composition of the Tyrode solution.

#### Constant-pressure Langendorff mode

The effects of blebbistatin in hearts that were unloaded and perfused in constant-pressure Langendorff mode using our modified Tyrode solution and that of Fedorov *et al.* (2007) are illustrated in Fig. 8*C* (*n*= 5 for each group). The MAPD was significantly (*P* < 0.001) increased during blebbistatin perfusion irrespective of the Tyrode solution composition. Perfusion pressure was 59.4 ± 3.5 mmHg (our modified Tyrode) and 55.1 ± 4.3 mmHg (Fedorov Tyrode), whilst blebbistatin significantly (*P* < 0.05) reduced coronary flow irrespective of the composition of the Tyrode solution, from 47.5 ± 3.3 to 25.1 ± 5.3 ml min^−1^ (our modified Tyrode solution) and from 43.9 ± 1.6 to 31.2 ± 3.9 ml min^−1^ (Fedorov Tyrode solution).

#### Dose–response relationship

**Figure 9:**
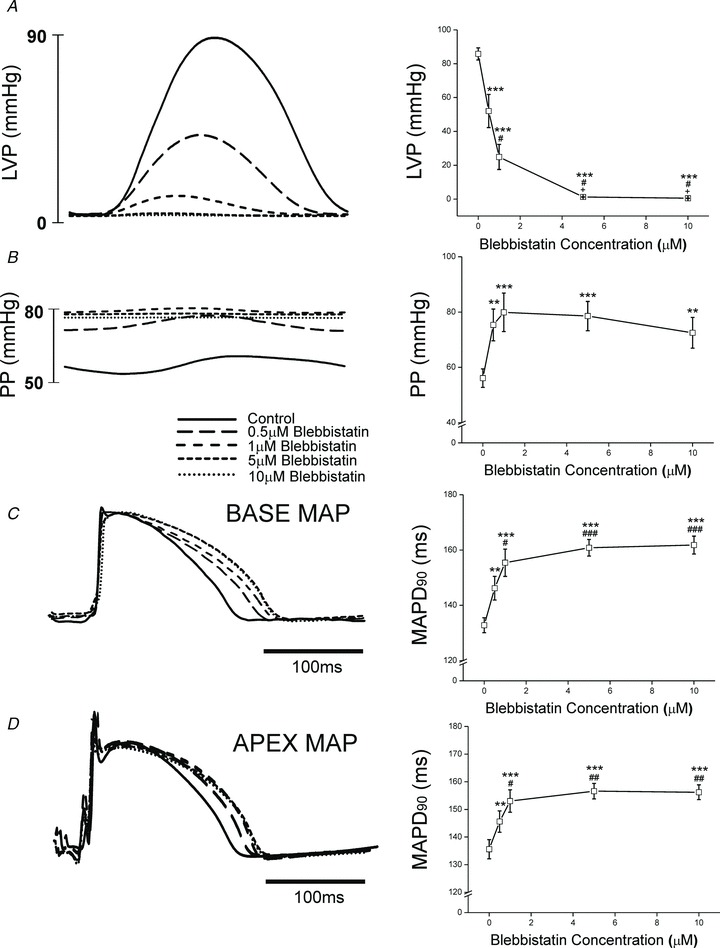
The dose–response effect of blebbistatin Left panels show aligned and averaged profiles for left ventricular pressure (LVP; *A*), perfusion pressure (PP; *B*), basal (*C*) and apical monophasic action potential duration (*D*) with 0.0, 0.5, 1, 5 and 10 μm blebbistatin perfusion. Right panels show mean data corresponding to each parameter. *n*= 6. *P < 0.05, **P < 0.01, ***P < 0.001 *versus* control. #*P* < 0.05, ##*P* < 0.01, ###*P* < 0.001 *versus* 0.5 μm blebbistatin. +*P* < 0.05 *versus* 1.0 μm blebbistatin.

The steady-state dose–response effect of blebbistatin is illustrated in Fig. 9. As shown in Fig. 9*A*, LVP was significantly decreased with a blebbistatin concentration as low as 0.5 μm, with a complete abolition of contraction at 5 μm. There was a significant increase in PP from doses of blebbistatin of 0.5 m and above (Fig. 9*B*). These data demonstrate a clear and significant vasoconstriction in response to blebbistatin. However, we observed that, compared with the level of PP during perfusion with 5 μm blebbistatin, perfusion with 10 μm blebbistatin resulted in an identifiable vasodilatation, i.e. perfusion with 10 *versus* 5 μm only. The PP during 10 μm was, however, still significantly higher than the control value. With respect to apical and basal MAPD, blebbistatin produced a significant prolongation in MAPD from 0.5 μm in a dose-dependent manner (Fig. 9*C* and *D*).

### Optically recorded action potentials

**Figure 10:**
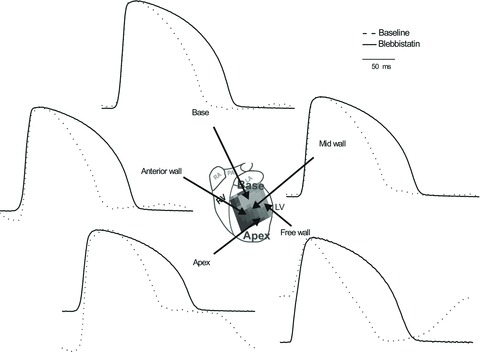
Effect of blebbistatin on optical mapping recorded action potentials Illustration of optically recorded action potentials using di-4-ANEPPS at base, mid-wall, free wall, apex and anterior wall sites at baseline and after perfusion with 5 μm blebbistatin during right ventricular pacing (300 ms cycle length).

The effect of blebbistatin was investigated using optically recorded action potentials in four hearts and is illustrated in Fig. 10. There was a clear prolongation of action potential duration. Attempts were made to quantify multisite APD; however, owing to significant motion artifacts present prior to the use of blebbistatin, which can be observed especially at the free wall and apical sites, accurate APD could not be obtained from all sites. There was, however, a stable action potential recording from the centre of the recording area corresponding to the mid-wall region of the left ventricle. At this site, baseline APD was measured at 136.5 ± 6.3 ms and was significantly increased to 173.0 ± 7.9 ms in the presence of blebbistatin (*P* < 0.05, *n*= 4). This equated to a mean increase of 36.5 ± 10.3 ms, or 27.6 ± 8.7%. In addition, baseline APD and APD measured during blebbistatin perfusion were remarkably similar when using either contact electrodes or optically recorded techniques, suggesting that regardless of the recording technique, blebbistatin significantly prolongs action potential duration.

## Discussion

In this study, the effects of the ECU blebbistatin on LVP, PP, atrioventricular conduction, MAPD, QT interval, standard APD restitution and VF inducibility were measured. We have shown that blebbistatin resulted in an expected abolition of ventricular contractile force production and a biphasic effect on PP, with an initial vasodilatation followed by a secondary increase in PP with concomitant reduction in coronary flow. Blebbistatin did not affect heart rate, atrioventricular conduction or intraventricular conduction. However, we have shown, for the first time, that ventricular MAPD is prolonged, mirrored by QT interval prolongation, and the APD restitution slope and VF threshold are both significantly increased during perfusion with blebbistatin. Finally, we observed a consistent increase in MAPD in conditions of constant pressure and constant flow, in hearts that did not have any load in the left ventricle and in action potentials recorded with optical mapping. These findings are important with regard to their impact upon the field of optical mapping and suggest that further confirmatory study is required, as well as investigations into the mechanism of action.

### Disparity with previous blebbistatin studies in cardiac models

The changes observed in this study differ from those previously reported. Fedorov *et al.* (2007) assessed the effects of blebbistatin on cardiac electrophysiology in isolated, Langendoff-perfused rabbit hearts, isolated ventricles, isolated atria and single rat myocytes. They found that blebbistatin did not significantly alter action potential morphology, nor did it affect electrical patterns or refractory periods. These authors also showed, in single rat ventricular myocytes, that blebbistatin had no effect on intracellular calcium handling; however, this was not investigated in the whole heart. Jou *et al.* (2010) also showed that blebbistatin, in embryonic zebrafish hearts, had no significant effect on action potential morphology, repolarization time, ERP or upstroke velocity.

To our knowledge, the present study is the first to demonstrate that blebbistatin causes significant changes in the ventricular electrophysiology, although Kanlop & Sakai (2010) noted spontaneous electrical activity in response to high concentrations of blebbistatin (10–100 μm). We did not demonstrate similar spontaneous activity in our experiments; however, the dose required to abolish motion artefacts (5 μm) is lower than those reported by Kanlop & Sakai (2010). Nevertheless, these data are worthy of note and are of potential importance.

Currently, we have little explanation regarding the difference between our observations and previous reports, despite performing extensive control experiments to address differences in experimental approach, which includes replication of experimental conditions used in other studies. It is important to highlight that all hearts within our study were allowed to equilibrate for a period of 30 min prior to baseline measurement. Once a baseline was achieved, our measurements of MAPD, electrical restitution and VFT remain stable within an acceptable range over a period of up to 5 h, as indicated in the control stability data presented within this manuscript (Fig. 1). We are therefore confident of the viability of our heart preparations over the experimental time frame. Moreover, it seems likely that VF would be easier to induce in a deteriorating preparation, which contrasts with the increase in VFT demonstrated in the present study. We have performed additional experiments to support out findings of MAPD prolongation using volume-conducted ECGs. These data show no evidence of ST segment elevation or depression, either at baseline or during perfusion with blebbistatin, which would be indicative of myocardial ischaemia. More importantly, the QT interval was prolonged to a similar extent with blebbistatin, as with our data on MAPD. Further to this, the prolongation in MAPD occurred in a dose-dependent manner, with significant MAPD prolongation occurring at a concentration of blebbistatin as low as 0.5 μm.

### Methodological considerations

In considering the differences between the findings of the present study in comparison to previous reports, we have performed control experiments to explore methodological differences between our study and others, which are discussed below, including the method of Langendorff perfusion, intraventricular load, composition of the perfusate, the method of measuring action potentials and recirculation of the perfusate.

#### Langendorff perfusion

The method of Langendorff perfusion is worthy of some discussion. Data published by Fedorov *et al.* (2007) were obtained from hearts perfused in a constant-pressure mode, which differs from the majority of data in the present study, collected in conditions of constant flow. In the present study (constant-flow) perfusion pressure was initially and significantly decreased during blebbistain perfusion (5 μm), in keeping with its known vasodilatory properties (Eddinger *et al.* 2007). This was, however, followed by an unexpected secondary and prolonged increase in PP, consistent with coronary vasoconstriction. During the dose–response experiments, a more prolonged vasodilatory effect was seen during high-concentration blebbistatin perfusion (10 *versus* 5 μm), though PP was still significantly greater than control values. This trend was not found to be significant.

In principle, this vasoconstriction could lead to a degree of mild global cardiac ischaemia, which may have secondary effects on cardiac electrophysiology, in addition to promoting tissue oedema. Such secondary ischaemia could cause a shortening of MAPD (Sutton *et al.* 2000), which could counterbalance MAPD prolongation, as seen in our study. In this regard, blebbistatin-associated vasoconstriction would lead to reduced coronary flow in conditions where coronary PP was held constant, as in previous studies utilizing Langendorff-perfused rabbit hearts with PP maintained at 60 ± 5 mmHg (Fedorov *et al.* 2007; Lou *et al.* 2012). The resultant alterations in coronary flow could cause a degree of ischaemia when flow is reduced, or promote an increased risk of cardiac oedema when perfusion flow is significantly increased. Either way, cardiac electrophysiology would be affected by variations in coronary flow and could potentially mask underlying effects of blebbistatin. It is important to note that previous studies using blebbistatin have not reported effects on coronary flow and perfusion pressure, which might reveal changes in vascular resistance to support our data. This has implications for all studies using blebbistatin.

Nevertheless, the energetic demand of the uncoupled heart is predictably less than that of a contracting heart, and it is possible that the decrease in flow is not sufficient to promote ischaemia. In support of this, Swift *et al.* (2012) have demonstrated that NADH accumulation in ischaemic tissue is five times slower in the presence of blebbistatin than that seen in control conditions. It cannot be ruled out that vasoconstriction is a reflection of a reduction in energetic demand. It has been proposed that coronary vascular tone is regulated primarily by metabolic factors, such as adenosine (Camici & Crea, 2007). When energy supply is high and/or energy demand low, the metabolic vasodilatation of the terminal arteriolar bed will be reduced, leading to vasoconstriction. As a consequence, vessel shear stress in larger coronary arteries will be reduced, decreasing NO-dependent vasodilatation. The net effect of these changes will be a reduction in coronary flow and an increase in coronary resistance and PP. It is attractive to hypothesize that a reduction in energetic demand during blebbistain perfusion drives vasoconstriction through this mechanism.

Researchers in our laboratory (Patel *et al.* 2008) and other groups (Stowe *et al.* 1997) have previously demonstrated that the uncoupling agent 2,3-butanedione monoxime has a significant vasodilatory action that contrasts with the effects of blebbistatin seen in the present study. It is, however, recognized that 2,3-butanedione monoxime has additional metabolic effects. 2,3-Butanedione monoxime may increase the rate of ATP hydrolysis, as well as impairing ATP transport between the mitochondria and cytosol, reducing ATP availability despite inhibition of contractile activity and a reduction in net oxygen consumption (de Tombe *et al.* 1992; Stapleton *et al.* 1998).

Swift *et al.* (2012) have previously reported that blebbistatin can form a precipitate when mixed in solutions at room temperature and can accumulate within the coronary vasculature. This is unlikely to be a factor in the present experiments, because all solutions were heated to 37°C and passed through a 5 μm micropore filter before perfusion.

The blebbistatin-mediated increase in PP seen in our study appears at odds with previous work using isolated arterial smooth muscle showing that blebbistatin inhibits contraction in isolated smooth muscle preparations (Eddinger *et al.* 2007). This suggests that blebbistatin should decrease PP. In our study, there was both a slowly developing increase in PP and a small initial vasodilatory effect, which may indicate two distinct phenomena. Data collected from hearts perfused in constant-pressure mode demonstrate a marked reduction in coronary flow, in support of our initial findings. Despite the reduction in flow, there was still a marked prolongation of APD during blebbistatin perfusion. Irrespective of the perfusion method used, blebbistatin still directly affected ventricular electrophysiology.

#### Intraventricular load

Studies employing optical mapping frequently do not record intraventricular pressure with a balloon or have any intraventricular load within the left ventricle, i.e. the ventricle is unloaded. The effects of ventricular stretch during loading could potentially affect APD (Franz *et al.* 1989). We undertook a series of experiments to investigate the potential role of ventricular load in the discrepancies between the present and previous studies. A similar degree of APD prolongation was seen in unloaded and loaded hearts, and ventricular load had no effect on APD in control conditions and during blebbistatin perfusion.

#### Composition of the Tyrode solution

In the present study, we used two compositions of Tyrode solution, one that is used usually in our laboratory and the other that was identical to that of Fedorov *et al.* (2007). A significant difference was a lower Ca^2+^ concentration of 1.3 mm in the solution of Fedorov *et al.* (2007) compared with 1.8 mm in ours. The Ca^2+^ concentration significantly affects APD, with decreasing external [Ca^2+^] significantly prolonging APD (Ricco *et al.* 2009; Brack *et al.* 2010). Despite this, when we mimicked the experimental conditions of Fedorov *et al.* (2007) (i.e. using the same chemical composition of Tyrode solution, constant-pressure Langendorff perfusion and unloaded ventricle), we still saw a significant prolongation of MAPD.

#### Recording methodology for APD

Other studies have used different recording techniques, including optical mapping, which allow conduction times to be determined on whole heart and isolated atrial and ventricular preparations before and after blebbistatin perfusion; in addition, APD data were also acquired using microelectrode techniques on atrial and ventricular preparations (Fedorov *et al.* 2007). When we examined the effect of blebbistatin in four hearts using optical mapping, significant increases in APD were still seen. It is noteworthy that in these conditions, the degree of APD prolongation with blebbistatin was similar across all experimental groups. More recently, Lou *et al.* (2012) recorded monophasic action potentials using contact electrodes whilst performing an extrastimulus protocol, i.e. electrical restitution. In their study, MAPD was not affected using 10 μm, which is at odds with the present study. However, despite performing this protocol, electrical restitution curves were not reported, so we cannot comment on whether MAPD at the shortest diastolic intervals was altered, which might have an effect on the slope of the restitution curve. In summary, irrespective of the experimental conditions that were tested, we found that blebbistatin consistently prolonged ventricular action potential duration.

#### Recirculation

Blebbistatin is expensive, which has led investigators to use a recirculating approach during perfusion. With this approach, the coronary effluent is collected and recirculated into the coronary circulation over the course of the experiment. Substances released from the heart during contraction, from the action of shear stress of the coronary endothelium and byproducts from metabolism, would be collected within the coronary effluent. One such substance is adenosine, which would promote vasodilatation and directly affect electrophysiology (Mustafa *et al.* 2009). Over time, these chemicals will accumulate upon repeated recirculation. This approach could provide a continually changing environment that may not only have a significant effect on cardiac electrophysiology but may also mask underlying effects of blebbistatin. Owing to these limitations, we avoid this practice within our laboratory. Despite the higher cost, all experiments in the present study were carried out with single pass and filtered continuous perfusion of blebbistatin.

### Action potential duration restitution and ventricular fibrillation

Several of the studies mentioned have attempted to determine the effects of blebbistatin on intracellular calcium handling. Fedorov *et al.* (2007) used a non-ratiometric calcium indicator, fluo-5F, to determine calcium transients in isolated rat ventricular cells, and they found that although calcium transients were not affected, the resting diastolic fluorescence increased. The authors suggested that this may be due to either the release of calcium into the cytoplasm from myocardial relaxation, or due to the photosensitivity of blebbistatin. Farman *et al.* (2008) used indo-1 in isolated rat ventricular cells and showed that blebbistatin did not affect calcium handling. Dou *et al.* (2007) used mouse myocytes and showed that there was no significant change in action potential duration with blebbistatin, and that there was no alteration in calcium transients through L-type calcium channels, despite the significant inhibition of myocyte shortening.

The mechanism suggested by Fedorov *et al.* (2007), by which calcium is released into the cytosol, may be a plausible explanation for the APD prolongation we observed, because this increased intracellular calcium would lead to an increase in the calcium transient. The spontaneous electrical excitations observed in the study by Kanlop & Sakai (2010), albeit at higher blebbistatin concentrations (10–100 μm), also suggest altered intracellular calcium handling.

An interesting finding in our study was the dissociation of the electrical restitution property from ventricular fibrillation inducibility. Our previous data on restitution slope and VFT support an inverse relationship between the two parameters, especially in conditions of autonomic stimulation, which is in agreement with the restitution hypothesis, i.e. increase in restitution slope and increase in VF susceptibility (reduction in VFT). In the present study, we showed dissociation between the two parameters, with an increased VF threshold (reduced susceptibility to VF) coupled with an increase in restitution slope following blebbistatin perfusion, suggesting that other arrhythmogenic mechanisms may be relevant. The mechanism underlying these results would need further investigation. However, an increase in ERP was observed, which may be associated with the increased VF threshold despite an increase in restitution slope.

Another noteworthy finding in our study was that blebbistatin prolonged S2 delay, suggesting a decrease in this surrogate marker of conduction velocity, although this could also represent delayed activation. We did not observe any change in pacing threshold during the experiment or during blebbistatin perfusion. An alternative hypothesis is that conduction slowing may reflect the encroachment of the S2 wavefront onto myocardium that is not fully repolarized, owing to the significant prolongation of MAPD, as opposed to a direct slowing of conduction. Nevertheless, it should be considered that the prolongation in S2 delay may represent an additional mechanism that causes the increase in VFT following blebbistatin perfusion.

Monophasic action potentials were measured from only two sites of the left ventricular epicardial surface; therefore, the contribution of dispersion of repolarization (transmural or over the epicardial surface) was not assessed, which may have an additional impact on arrhythmogenesis (Nicolson *et al.* 2012). The present study was aimed at obtaining data on the effect of blebbistatin on ventricular electrophysiology at two epicardial sites, to form a basis from which studies on potential mechanisms and other electrophysiological effects can be developed.

### Implications and further work

Although several previous studies suggested the absence of changes in intracellular ionic currents with blebbistatin, our findings suggest that changes may occur. Although blebbistatin is regarded as the ECU of choice, the associated electrophysiological effects have significant implications for data collected during optical mapping studies. The majority of previous work was carried out in cellular models. Given that the changes we observe are seen in a Langendorff-perfused whole heart preparation, our future studies will be aimed at determining the effects of blebbistatin on intracellular calcium handling using calcium-sensitive dyes in isolated hearts to add insight to the observations seen. Alternative mechanisms of action, including direct modulation of individual ionic currents, will also be investigated.

### Limitations: contact MAP electrode methodology

Contact MAP electrodes were used for this study, and it is accepted that these can be susceptible to motion artifacts. These artifacts usually manifest as deflections that are not consistent with what would be expected physiologically. Examples include sporadic unexpected deflections or ‘bumps’ during the cardiac cycle, partial negative recordings during the diastolic interval, or effects on the upstroke. Monophasic action potentials are extracellularly recorded signals, which reflect characteristics of intracellular action potentials and accurately report action potential duration.

Although MAPD can be used to provide temporal information on action potential duration, the amplitude of the MAP signal bears little relationship to the membrane action potential, which is why MAP amplitude is defined as the difference between the baseline and the crest of the plateau phase. The upstroke of the MAP signal represents the voltage difference over time in the small space (2 mm) between the recording and indifferent electrodes, and the intrinsicoid deflection represents the timing of local activation at the recording electrode, regarded as the beginning of the MAP signal relative to which duration is measured. In our study, MAPs could exhibit a phase 1-like morphology and ‘notch’ during the upstroke, representing an intrinsicoid deflection. This has been reproduced by others (Zabel *et al.* 1995; Franz, 1999, 2003; Kondo *et al.* 2004) and is widely seen in studies using the Franz contact electrode. These deflections are evident both at baseline when the heart is contracting and during blebbistatin perfusion when motion is abolished. This suggests that the MAP morphologies as shown in the present study are not influenced by myocardial contraction and perhaps represent the detection of activation as it moves beneath the recording electrode. Our finding that blebbistatin prolongs MAPD cannot be explained by motion artifact due to contraction.

### Conclusions

In isolated, Langendorff-perfused rabbit hearts, blebbistatin (5 μm) caused significant changes in cardiac function, including an initial decrease and secondary increase of perfusion pressure, prolongation of action potential duration, increase of electrical restitution slope and shift of ventricular fibrillation threshold. Some of these changes were not completely reversed following a 2 h period of washout with blebbistatin-free Tyrode solution.
